# Real-time observational evidence of changing Asian dust morphology with the mixing of heavy anthropogenic pollution

**DOI:** 10.1038/s41598-017-00444-w

**Published:** 2017-03-23

**Authors:** Xiaole Pan, Itsushi Uno, Zhe Wang, Tomoaki Nishizawa, Nobuo Sugimoto, Shigekazu Yamamoto, Hiroshi Kobayashi, Yele Sun, Pingqing Fu, Xiao Tang, Zifa Wang

**Affiliations:** 1 Institute of Atmospheric Physics/Chinese Academy of Sciences, State Key Laboratory of Atmospheric Boundary Layer Physics and Atmospheric Chemistry, Beijing, 100029 China; 2 Research Institute for Applied Mechanics, Kyushu University, Kasuga, Fukuoka, 816–8580 Japan; 3 National Institute for Environmental Studies, Tsukuba, Ibaraki, 305–8506 Japan; 4 Fukuoka Institute of Health and Environmental Sciences, Daizaifu, 818–0135 Japan; 5 University of Yamanashi, Yamanashi, 400–0016 Japan

## Abstract

Natural mineral dust and heavy anthropogenic pollution and its complex interactions cause significant environmental problems in East Asia. Due to restrictions of observing technique, real-time morphological change in Asian dust particles owing to coating process of anthropogenic pollutants is still statistically unclear. Here, we first used a newly developed, single-particle polarization detector and quantitatively investigate the evolution of the polarization property of backscattering light reflected from dust particle as they were mixing with anthropogenic pollutants in North China. The decrease in observed depolarization ratio is mainly attributed to the decrease of aspect ratio of the dust particles as a result of continuous coating processes. Hygroscopic growth of Calcium nitrate (Ca(NO_3_)_2_) on the surface of the dust particles played a vital role, particularly when they are stagnant in the polluted region with high RH conditions. Reliable statistics highlight the significant importance of internally mixed, ‘quasi-spherical’ Asian dust particles, which markedly act as cloud condensation nuclei and exert regional climate change.

## Introduction

Mineral dust is considered one of the key contributors to global aerosol loadings^1, 2^, and it disturbs the atmospheric radiative balance^3, 4^, affects nutrient supplies in the marine ecosystem^5, 6^, and superimposes detrimental health impacts^7^. In East Asia, industrial/polluted region is located on transport pathway of dust plume^8^. Dust particles normally mixed with substantial amounts of pollutants and served as a good carrier for long-range transport of anthropogenic pollutants. Numbers of previous studies using off-line sampling and electron microscopy inspection reported a visible coatings (i.e. sulfate, nitrate) on some individual dust particles that were collected not only in the polluted area^9, 10^ but also in the remote maritime environment^11^. A high spatial specificity for the composition and structure of dust particles with different degrees of aging has been archived^12, 13^. Nevertheless, the off-line based analysis requires manual operation and is labor-intensive, which results in a relatively large uncertainty and poor statistics^14^. The depolarization ratio (defined as the ratio of *s*-polarized to *p*-polarized signals; see Method) of the oscillation direction of the electromagnetic wave of scattering light from the illuminated particle is an applicable surrogate to indicate the irregularity of the dust particles^15, 16^. Theoretical calculation on ellipsoid particles indicated that depolarization ratio of particle decreased evidently as its aspect ratio (defined as ratio of the longest dimension to its orthogonal width) decrease, which provided the possibility to investigate the time-resolved morphological variability of dust particles as it mixed with anthropogenic pollutants. Lidar (acronym of Light Detection And Ranging) observation is a powerful tool and provides the overall aerosol depolarization of an aggregation of multiple particles at high temporal/spatial resolution^17, 18^, however its volume scattering measurement cannot distinguish the internal/external mixing state of particles clearly^19^. Therefore, the *in-situ* and real-time measurement of the polarization change of single particles is necessary to obtain a better understanding of the mixing state of the dust particles, and there is no longer a need to presume the size distribution and morphology of the particles in optical models. A bench-top optical particle counter equipped with a depolarization module (Polarization Optical Particle Counter; POPC)^20^ was recently developed and provides a size-resolved polarization study for individual particles^21, 22^. Till now, there was no real-time measurement of morphological variations of dust particles in Beijing mega city, and the effect of neither chemical composition nor meteorology has been clarified.

A typical floating dust event was observed in North China on March 28–31, 2015. The main part of the dust plume originated from central Inner Mongolia and first arrived at a downtown area of Beijing mega city (observation site) on March 28. It was transported southerly to Shandong Province and dispersed into two parts (March 29). The northern half of the dust plume returned back and was stagnant over the Beijing area for approximately 3 days. This made the continuous observation of the same dust plume throughout the event possible. During dust event, we performed comprehensive measurements of the size distribution, polarization properties and chemical composition of aerosol particles. The purpose of this study is to quantitatively investigate morphological evolution of dust particles as mixing with pollutants, and to quantify the impact of both water-soluble inorganic (i.e. nitrate) and relative humidity (RH) on the formation of spherical dust particle in polluted area.

## Results

**Figure 1:**
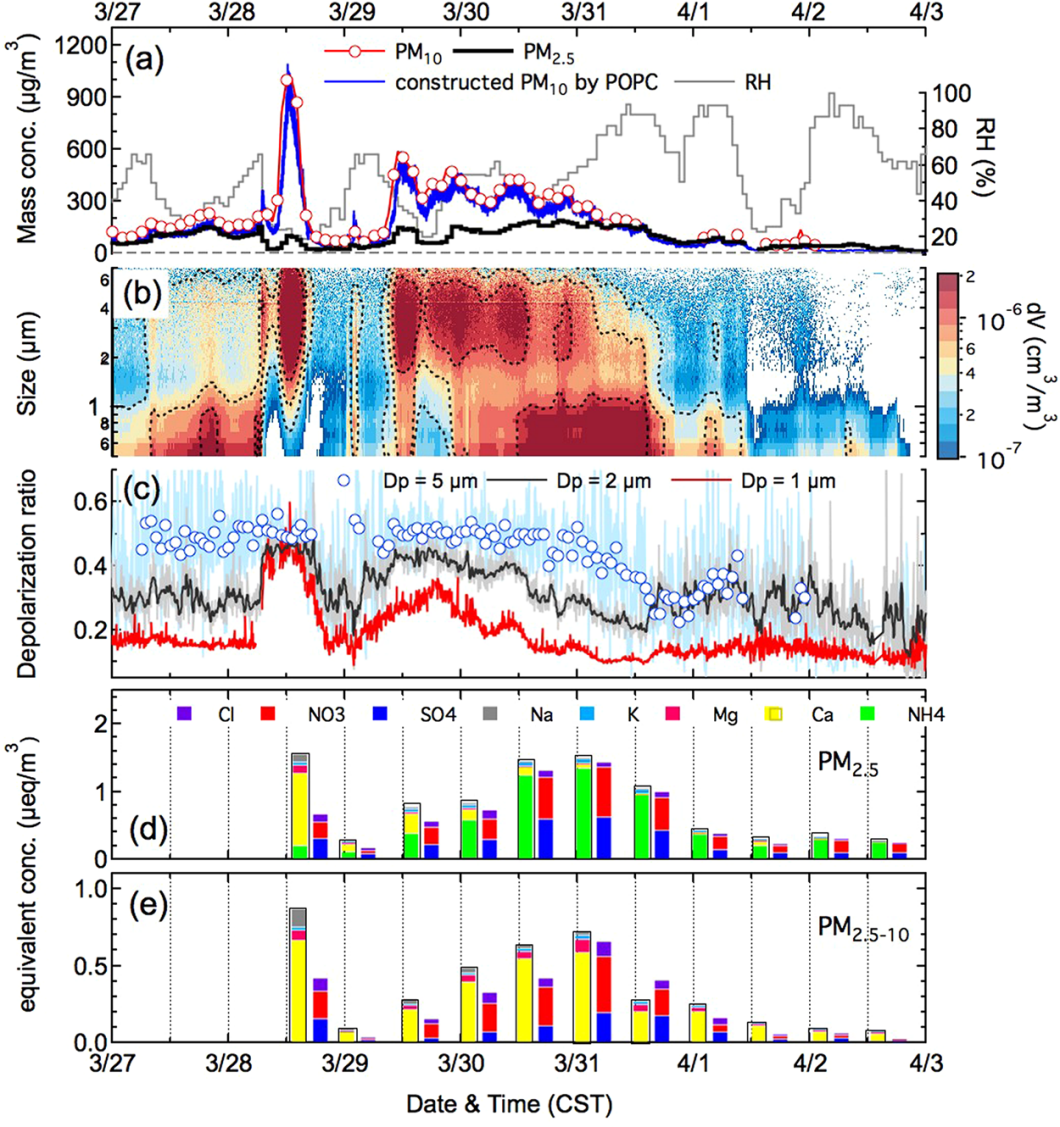
Time series of the observed mass concentration of PM_2.5_, PM_10_ and constructed PM_10_ according to the POPC measurement, relative humidity (**a**), volume size distribution (**b**), hourly averaged depolarization ratio at Dp = 1 μm, 2 μm and 5 μm (**c**) and equivalent concentrations of identified water-soluble compounds in both the fine mode (PM_2.5_) (**d**) and coarse mode (PM_2.5–10_) (**e**) on the basis of filter-based chromatography analysis.

The main dust plume first arrived at Beijing at 1200 CST (China Standard Time) on March 28, 2015. Mass concentrations of PM_2.5–10_ (aerodynamic diameter of particles between 2.5 μm and 10 μm) increase sharply to 1049 μg/m^3^, about tenfold that of PM_2.5_ (aerodynamic diameter of particles less than 2.5 μm, 103 μg/m^3^) (Fig. 1a and Fig. 1b). We selected particles at an optical size (D_p_) of 5 μm to represent the dust particles^8, 23^, and fine mode particles at D_p_ = 1 μm for anthropogenic pollutants as references. During the onset of dust event, the uncontaminated dust particles were found to have a mean depolarization ratio of 0.5 ± 0.02 (Fig. 1c). The hourly averaged depolarization ratios of the particles at D_p_ = 1 μm increased significantly to 0.45 ± 0.01 due to the influence of fine non-spherical dust particles (Fig. 1c). With the transport of dust plume, mass concentration of PM_2.5–10_ decreased significantly due to gravity settlement velocity and dilutions, whereas mass concentration of PM_2.5_ gradually increased to 148 μg/m^3^ at the end of the dust episode on March 31, as a result of production of secondary pollutants. The hourly averaged depolarization ratio of the dust particles decreased by approximately 46%, with a mean of 0.34 ± 0.05, as dust particles were continuously mixed with secondary pollutants. Depolarization ratios of the particles at D_p_ = 1 μm and D_p_ = 2 μm decreased to their reference values of 0.1 ± 0.01 and 0.2 ± 0.03, respectively (Fig. 1c).

**Figure 2:**
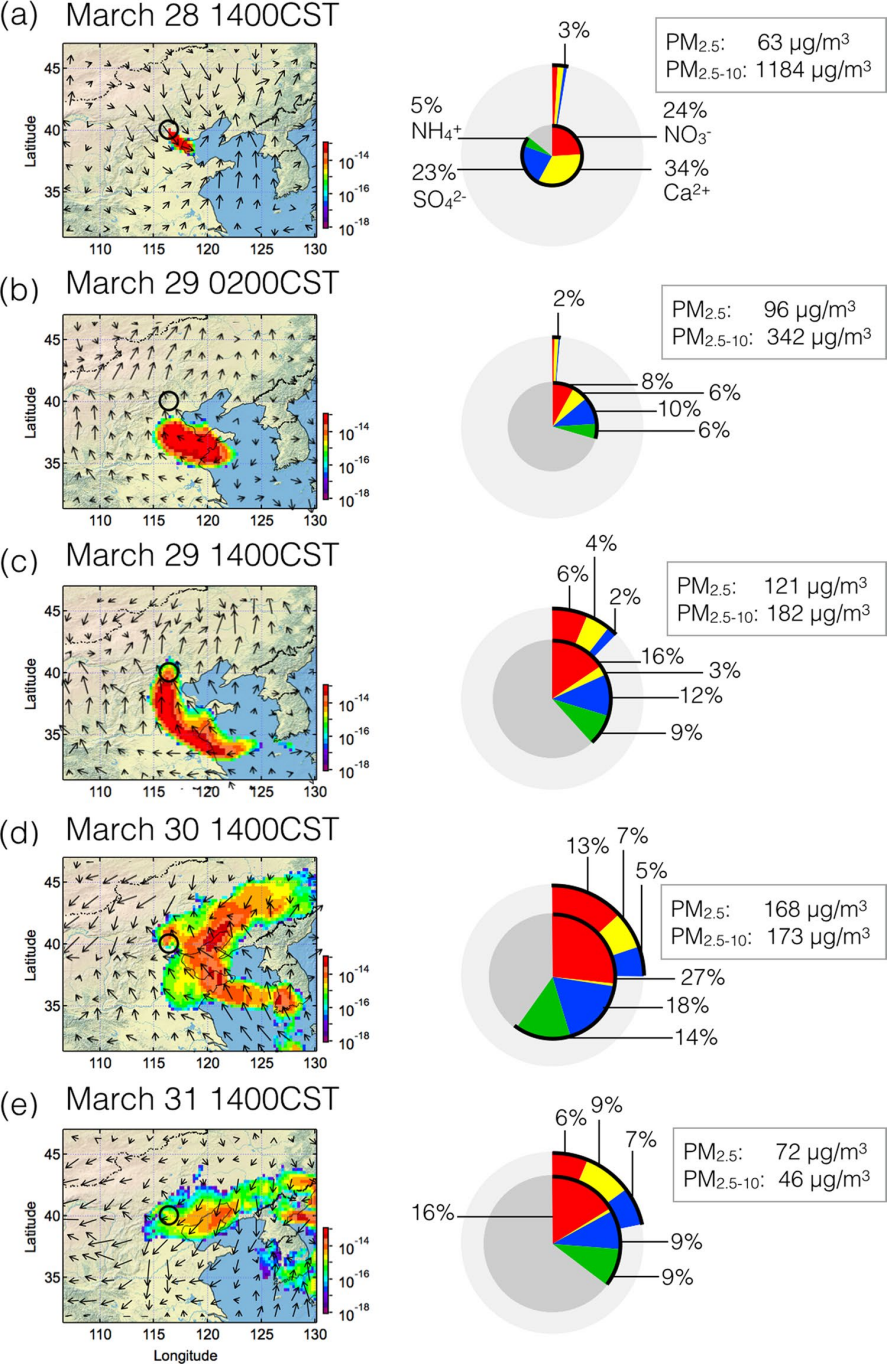
Footprint of the dust plume simulated by the HYSPLIT dispersion model ((**a**) detailed description of the simulation is shown in **Method**) during the dust-influencing period (on the left). The tracer particles were released from 500–1000 m above ground level at the observation site and dispersed for 5 days. The mass concentration of the particle (in mass/m^3^) ranged from 0 to 1000 m every 3 hours. The figures on the right show corresponding mass concentrations of water-soluble inorganic matter (SO_4_^2−^: blue; NO_3_^−^: red; NH_4_^+^: green; and Ca^2+^: yellow) in total PM_2.5_ (inner Pi-chart) and PM_2.5–10_ (outer gray ring). The maps were drawn by the software Igor Pro, http://www.wavemetrics.com/.

The equivalent concentration of water-soluble compositions on the filter samples in PM_2.5_ (Fig. 1d) and PM_2.5–10_ (Fig. 1e) showed that the ion balances were well achieved for nearly the entire period, except for the first onset of dust on March 28 when 97% of the particle mass in PM_2.5–10_ was related to crustal matters with mass fraction of water-soluble Ca^2+^ (cCa^2+^) less than 1% (Fig. 2a). On March 29, when the dust plume returned back to Beijing area (Fig. 2c), the mass concentrations of both the NO_3_^−^ (cNO_3_^−^: 11.6 μg/m^3^) and cCa^2+^ (7.8 μg/m^3^) in PM_2.5–10_ increased, suggested that the nitrate already started to accumulate due to absorption of nitric acid (HNO_3_) on the surface of Ca-rich dust particles. In PM_2.5_, secondary formation of water-soluble inorganic matter accounted for 44% of total PM_2.5_, with high mass contributions of NO_3_^−^ (fNO_3_^−^: 18.7 μg/m^3^) and SO_4_^2−^ (fSO_4_^2−^: 14.0 μg/m^3^). After March 29 (Fig. 2d), the dust plume was stagnant over the Beijing area. NO_x_ emission in the city was mostly responsible for the significant enhancement of mass concentrations of fNO_3_^−^ (44.9 μg/m^3^). Rapid increase of fSO_4_^2−^ (30 μg/m^3^) may also attribute to preferential formation of sulfuric acid under the influence of both of NO_x_^24^ and dust^25^. In PM_2.5–10_, mass concentrations of cNO_3_^−^ (22.8 μg/m^3^) and cCa^2+^ (11.7 μg/m^3^) were also predominant. At the end of dust event on March 31 (Fig. 2e), the cCa^2+^ mass still accounted for 9% of total PM_2.5–10_ mass, though its mass concentration was decreased 3.9 μg/m^3^, higher than that of cNO_3_^−^ (2.9 μg/m^3^) and cSO_4_^2−^ (3.1 μg/m^3^). Substantial coexistence of cCa^2+^ and cNO_3_^−^ indicated of presence of deliquescent Ca(NO_3_)_2_, which formed by reaction between CaCO_3_ and HNO_3_ on the Ca-rich dust particles^26^. Almost no NH_4_^+^ (cNH_4_^+^) was found in PM_2.5–10_. It was reasonable that preferential formation of (NH_4_)_2_SO_4_ occurred at first in fine mode according to the good correlation (*r* ^2^ = 0.94) between SO_4_^2−^ and NH_4_^+^. Because the Asian dust included substantial amount of calcium^23, 27^, reaction between Ca(CO_3_)_2_ and (NH_4_)_2_SO_4_ on the surface of dust particles may result in the loss of NH_4_^+^ and the formation of insoluble CaSO_4_^28^.

**Figure 3:**
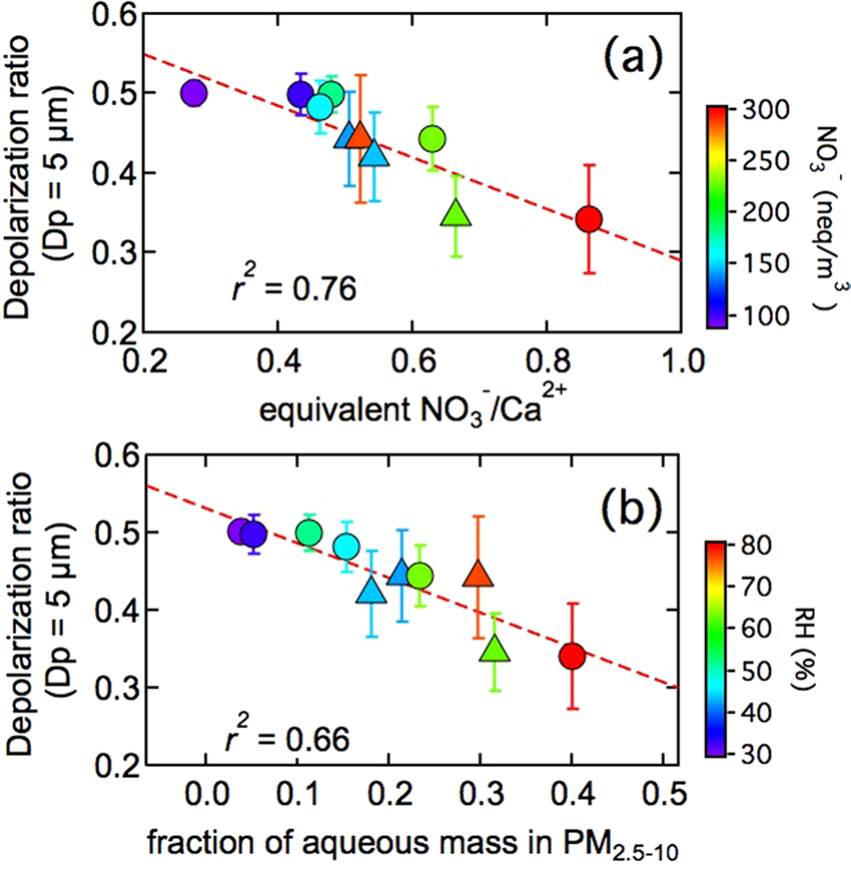
Relationship between the depolarization ratio of dust particles (Dp = 5 μm) and equivalent ratio of NO_3_^−^/Ca^2+^ (**a**) and the mass fraction of aqueous matter in the coarse mode (**b**). The colored circles in the plot represent the data during dust impact period from March 28 to April 1, 2015. The standard deviation (error bar) of depolarization ratio of dust particle was calculated for the dataset corresponding to filter sampling period. The color triangles indicate another weak floating dust case from April 9 to April 11, 2015. The backward trajectory analysis using HYSPLIT indicated that the air mass also came from northwest. Although none of the same dust plume was observed, the impact of anthropogenic pollutants on the depolarization ratio of the dust particle was similar.

Hourly averaged depolarization ratio of dust particles decreased as equivalent ratio of cNO_3_^−^/cCa^2+^ increased with a correlation coefficient *r* ^2^ = 0.76 (95% confidence interval, Fig. 3a). It indicated that, the more cNO_3_^−^ mass present in the coarse mode, the more possibility to lead to the morphological change of dust particles due to heterogeneous reaction between CaCO_3_ and HNO_3_ on the dust particles in polluted urban area^26^. The formation of Ca(NO_3_)_2_ coating on the surface of dust particles reduced the critical super-saturation of dust particles and have strong potential to serve as CCN^29^. Impact of Na^+^ (1.4%), Cl^−^ (0.8%) and K^+^ (1.3%) in PM_2.5–10_ on the decrease of depolarization ratio of dust particles were limited.

Morphological changes in dust particles were a synergistic effect of both pollutants and water content on the dust surface. Increase of relative humidity (RH) plays a vital role in decrease of the depolarization ratio of the dust particles in the presence of Ca(NO_3_)_2_ coating. The correlation coefficient between the depolarization ratio of the dust particles and the mass fraction of the aqueous content (water + Ca^2+^  + NO_3_^−^) in PM_2.5–10_ was *r* ^2^ = 0.66 (95% confidence interval, Fig. 2b). Because all Ca(NO_3_)_2_ was deliquescent in the coarse mode when RH > 20% and underwent a exponential increase^30^, the volume fraction of aqueous mass in the particles was calculated assuming density of dust particles of 2.5 g/cm^3^ ^31^. From March 29 to March 30, the ambient RH was 48 ± 9%, the aqueous mass was estimated to be 28.5 ± 10.8 μg/m^3^, accounting for 17% of total volume in PM_2.5–10_. However, at the end of dust episode on March 31, aqueous mass was 55.5 ± 13.7 μg/m^3^ on average, accounting for as high as 70% in the total volume of PM_2.5–10_ at RH = 86%. Large fraction of aqueous matter well explained the obvious decrease in depolarization ratio of dust particles.

**Figure 4:**
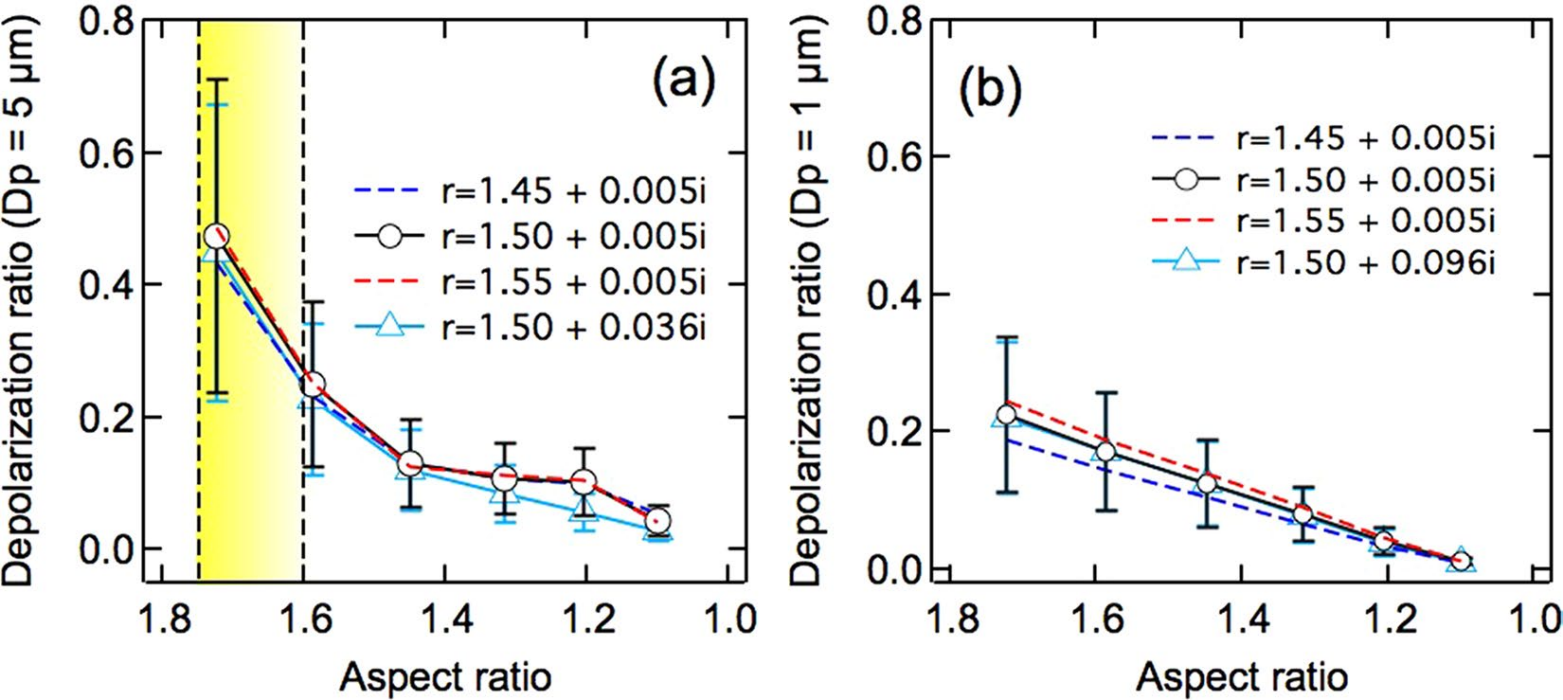
Theoretical simulation of the depolarization ratio of randomly oriented elongated ellipsoid particles as a function of the aspect ratio at Dp = 5 μm (**a**) and Dp = 1 μm (**b**) on the basis of the T-matrix methodology.

Although the observed depolarization ratio of the coated dust particles decreased evidently, we considered such dust particles as being ‘quasi-spherical’ because the observed minimal depolarization ratio (0.34) was still higher than that (0.08) of standard spherical particles (see Method). The polarization property of randomly oriented elongated ellipsoid particles was simulated on the basis of the T-matrix methodology^32, 33^. A reception angle of 120 degrees relative to its incident light direction was the same with the POPC instrumentation. Theoretical calculation revealed that the depolarization ratio of dust particles was mainly determined by its aspect ratio (defined as the ratio of the longest dimension to its orthogonal width). Variations in the particle's refractive index (the real and imaginary part) can only explain limited depolarization variability (5%). As indicated in Fig. 4a, the observed maximal depolarization ratio (0.5) in this study corresponded to an aspect ratio of 1.7 for uncoated dust particles. During the polluted dust period on March 31, the aspect ratios of the dust particles were estimated to be 1.6 as the depolarization ratio of the dust particles decreased to 0.34, as shown by the yellow shading in Fig. 4a. Providing that the dust particles were in a standard ellipsoid configuration and underwent partial hygroscopic growth only at the shortest projection, a 70% increase in the volume of coating matter on the dust surface would cause the aspect ratio to decrease, at most, from 1.7 to 1.3. We concluded that, the moderate decrease in aspect ratio of the dust particle demonstrated that the deliquescent and hygroscopic processes of Ca(NO_3_)_2_ occurred on the entire surface of the dust particles. The depolarization ratio of the particles with D_p_ = 1 μm showed a linear decrease with increase of aspect ratio (Fig. 4b).

## Discussion

Sulfate was frequently observed in the dust samples from Asian continent by electro-microscopy inspection^10, 13^. In this study, a negative correlation (*r* ^2^ = 0.75, 95% confidence interval) between depolarization ratio of dust particles and mass fraction of cSO_4_^2−^ in PM_2.5–10_ was found. The good equilibrium in PM_2.5–10_ implied that most of cSO_4_^2−^ in the sample may formed as CaSO_4_, the latter of which has small solubility (0.3%) and high deliquescent point (RH = 98%), and the large amount of cSO_4_^2−^ in PM_2.5–10_ was probably due to the significant dilution during the pretreatment of filter samples on the basis of ion chromatography analysis. In the real atmosphere, hygroscopic effect of CaSO_4_ on the dust morphology seemed to be negligible. One of the possible explanation is the heterogeneous reaction itself may modify non-sphericity of dust particles, As demonstrated in laboratory experiment, chemical processes of sulfuric acid (H_2_SO_4_) on dust directly led to evident dust surface modifications^34^; however direction conversion from CaCO_3_ to CaSO_4_ occurred more slowly and incomplete. Another contribution may rise from direct heterogeneous reaction between CaCO_3_ and (NH_4_)_2_SO_4_ at RH > 60% condition^28^. It was worthy noting that mass concentration of cNO_3_^−^ was much larger than cSO_4_^2−^ in dust plume, with a cNO_3_^−^/cSO_4_^2−^ mass ratio of 1.5–2.5, much higher than the value (0.16–0.5) reported ten years ago during the polluted dust period in Beijing^27^. It demonstrated that NO_x_ has exceeded SO_2_ as the most important pollutant in Beijing area, in accordance with bottom-up emission inventory estimation^35^. Consequently, more direct absorption of reactive HNO_3_ on the alkaline surface of dust particles becomes important to form strong hydrophilic compounds such as Ca(NO_3_)_2_^29, 36^, the latter of which absorb water vapors at relative humidity (RH) above 10%^30^, and apparently will affect to serve as cloud condensation nucleus (CCN).

Another important point, mineral dust and organic aerosols have traditionally been studied separately, however, more and more observations with a variety of means pointed out that adsorption of short-chain oxygenated hydrocarbons and low-vapor-pressure organic species (i.e. carboxylic acid) onto the dust surface cannot be overlooked^37–39^. For instance, shipboard aerosol time-of-flight mass spectrometer (ATOFMS) measurement of Asian aerosol outflow found that oxalic and malonic acids predominantly internally mixed with mineral dust, as a result of photochemical oxidation of volatile organic and diacids partitioning^40^. Quantitative analysis of organic acid-related coating on the morphological alteration of mineral dust was limited. In the present study, dependence of depolarization ratio on organic matter in PM_2.5–10_ remains unclear because of lacking of information of organic compound/function groups. During dust impact period, online measurement from Aerosol Chemical Speciation Monitor (Aerodyne Research, Inc.) at the site showed that mass concentration of organic matter accounted for ~40% of total mass in PM_1_, indicating of substantial formation of secondary organic aerosol. Redistribution of semi-volatile organics from submicron to dust particles was likely happened for some organic acid with lower vapor pressure, in particular under higher ambient RH condition. Recent observation study in western Japan^22^ using Aerosol Chemical Speciation Analyzer (Kimoto Electric Co., Ltd.) supported our speculations in this study, and pointed out that mass concentration of water-soluble organic carbon in coarse mode increased by a factor of three during a long lasting dust event.

This study, for the first time, investigated the real-time variation of depolarization property of dust particles as mixing with anthropogenic pollutants on the basis of newly developed polarization optical particle counter. The findings in this letter imply that the morphology of dust particles modified by internally mixing processes with water-soluble inorganic matters was statistically significant, and ‘quasi-spherical’ dust particle could substantially present in the polluted urban area, in particularly at high RH condition. The NO_x_ emissions in East Asia have been rapidly increasing over the last several decades^41^, and the impact of nitrate on the morphology of coarse mode mineral dust and its subsequent spatial allocation have become increasingly important. These findings also provide new motivation to revisit decades of Lidar data^18^ to provide a better understanding of the decadal variation in the changes in dust-pollution interactions against the background that anthropogenic emissions in East Asia have undergone profound changes^41^. This study also indicates the necessity of a reliable optical model of internally mixed polluted dust for a detailed analysis of polarization remote sensing observations.

## Methods

### Polarization Optical Particle Counter (POPC) and depolarization ratio

The POPC uses a 780 nm linearly polarized laser source and measures both forward and backward scattering intensity at 60 and 120 degrees relative to the direction of incident light. The polarization direction of the incident laser is parallel to the plane of the scattering angle. This configuration was optimized to reduce measurement uncertainty^20^. The depolarization ratio of the particles was defined as the ratio of the *s*-polarized to *p*-polarized signal (S/P) of the oscillation direction of the magnetic wave of scattering light from the particles. During the measurements, the pulse signals were sampled for 1 s and processed for 1.2 s. The sampling rate and half-width of full height (WHFH) of the POPC detector's output signal were 2 × 10^6^ samples/s and approximately 35 μs, respectively. To avoid the coincidence error of the measurements, the inlet flow rate of POPC was set to 0.08 liters per minute (lpm) and was diluted with zero air (0.92 lpm, RH = 38 ± 1%). The residence time of diluted air mass was estimated to be 0.7 s, which was generally sufficient for aerosol particles to achieve equilibrium before measurement in the detecting chamber. It suggested that the wet particles tend to shrink due to loss of water. The measurement uncertainty in size determination was estimated to be 10–15%. Provided that the sampled particles are homogeneously distributed, the upper detection limit was estimated to be 6 × 10^5^ particles per liter to fully meet the observation requirement. The optical size of the particle was converted from a forward scattering signal at 60 degree on the basis of the standard calibration curve, which was determined before the field campaign using standard spherical particles (Dynospheres, D_p_ = 0.5 μm, 1 μm, 3 μm, 5 μm, and 10 μm, JSR Life Sciences Corporation). Depolarization ratio of typical spherical particles at Dp = 3.344 μm (SS-033-P) and Dp = 5.124 μm (SS-053-P) were found to be 0.03 ± 0.01 and 0.07 ± 0.01, respectively. The instrument was installed at the top of a two-story building at the State Key Laboratory of Atmospheric Boundary Layer Physics and Atmospheric Chemistry (LAPC, Longitude: 116.3705E; Latitude: 39.9745 N), Institute of Atmospheric Physics/Chinese Academy of Sciences. Ambient air was drawn into the room through a 2-m-long vertical stainless steel tube (1/2 inch) with a laminar flow rate of 10 liters per minute. The turbulent loss of the coarse mode particles was limited.

### Footprint simulation by HYSPLIT Dispersion Model

HYSPLIT, which is short for Hybrid Single Particle Lagrangian Integrated Trajectory Model, is a Lagrangian transport and dispersion model that is suitable for simulating long-range atmospheric transport processes (http://ready.arl.noaa.gov/HYSPLIT_disp.php). In this study, the meteorological field was the GDAS (Global Data Assimilation System, NCEP) dataset with time intervals of 3 hours (observation data at 00, 06, 12, and 18 UTC and forecast data at 03, 09, 15, and 21 UTC) and a spatial resolution of 1 degree by 1 degree. The inert particles were released below 1000 m and above ground level for 3 hours. The dry deposition process (V_d_ = 0.5 cm/s) was considered during the simulation. The spatial distribution of the footprint region of the air samples was calculated on the 5 days of forward simulation for 1 unit mass of particles released between 500 and 1000 m above ground level at the observation site. The mass concentration of the particles (in mass/m^3^) at the surface was calculated by the HYPLIT dispersion model, considering large-scale convection, turbulent motions and the subgrid terrain effect in the simulation.

### Filter sampling and Chromatographyh

The filter sampling in both PM_2.5_ and PM_10_ were conducted on the LAPC campus from March 28 to April 12, 2015. Two aerosol samples were collected on quartz fiber filters (Pallflex) during both daytime (0800–1800 CST) and nighttime (1800–0800 CST) using a Hi-volume Air sampler (TISCH Environmental, Inc.) at a flow rate of 1 m^3^/minute. The sampled filters were placed in a constant temperature (25 °C) and humidity (40%) chamber for 48 hours and weighed in the laboratory. The mass concentrations of the sampled aerosols (e.g., PM_2.5_ and PM_10_) on the filter were obtained according to the weight difference before and after sampling. The sampled filters were cut into 2-cm-diameter circular specimens and dissolved in 10 mL of deionized water, followed by 10-minute mechanical vibration and 10-minute ultrasonic wave stirring. Then, the solution was analyzed by IC techniques for anions F^−^, Cl^−^, NO_3_^−^, and SO_4_^2−^ (ICS-1600, Thermo Fisher Scientific) and cations Na^+^, NH_4_^+^, K^+^, Mg^2+^, and Ca^2+^ (ICS-1100, Thermo Fisher Scientific). In particular, for all of the samples that were collected during heavy pollution periods, the solution was analyzed twice after undergoing dilutions of 2–5 times on the basis of the ambient mass concentration of PM_2.5_. Both of the IC analyses were used for final data quality assurance, and the mass concentrations of SO_4_^2−^, NO_3_^−^, NH_4_^+^, and Cl^−^ showed good correlation with an online Aerosol Chemical Speciation Monitor (ACSM) measurement as shown in *SF.1*.

## Electronic supplementary material

Supplementary Information
