# Supplementary material for: Real-time observational evidence of changing Asian dust morphology with the mixing of heavy anthropogenic pollution
Source: Sci Rep. 2017 Mar 23;7:335. doi: 10.1038/s41598-017-00444-w (PMC5428416; doi:10.1038/s41598-017-00444-w)
Supplement: Supplementary file 1 — Supplementary Information [file 41598_2017_444_MOESM1_ESM.pdf]

Supplementary information of

Real-time observational evidence of changing Asian dust morphology with the mixing of heavy anthropogenic pollution

Xiaole PAN[1,2,*], Itsushi UNO[2], Zhe WANG[1,2], Tomoaki NISHIZAWA[3], Nobuo SUGIMOTO[3], Shigekazu YAMAMOTO[4], Hiroshi KOBAYASHI[5], Yele SUN[1], Pingqing FU[1], Xiao TANG[1], Zifa WANG[1]

[1]Institute of Atmospheric Physics/Chinese Academy of Sciences, State Key Laboratory of Atmospheric Boundary Layer Physics and Atmospheric Chemistry, Beijing, 100029, China.
[2]Research Institute for Applied Mechanics, Kyushu University, Kasuga, Fukuoka, 816-8580, Japan
[3]National Institute for Environmental Studies, Tsukuba, Ibaraki, 305-8506, Japan
[4]Fukuoka Institute of Health and Environmental Sciences, Daizaifu, 818-0135, Japan
[5]University of Yamanashi, Yamanashi, 400-0016, Japan
*panxiaole@mail.iap.ac.cn

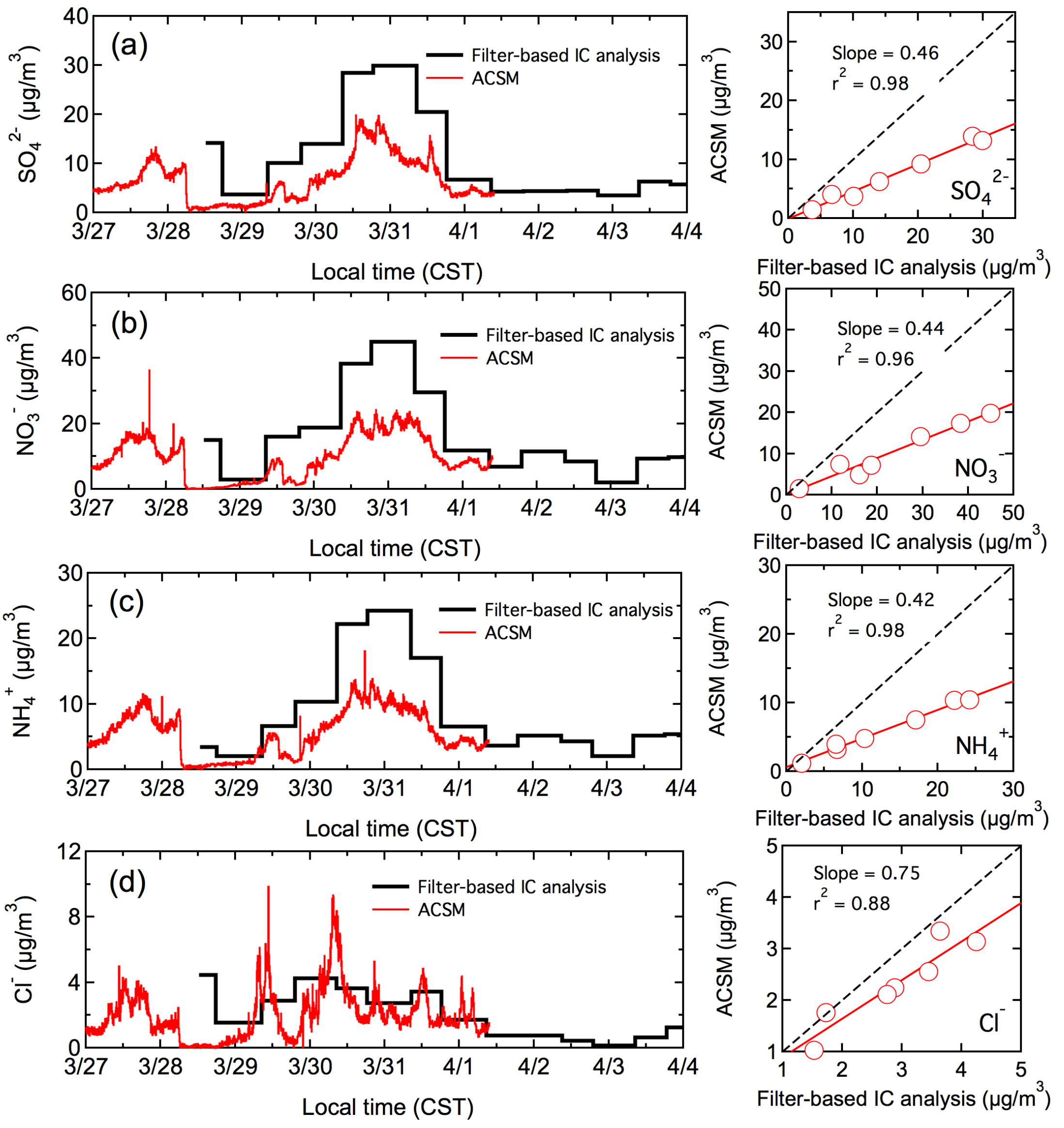

SF. 1 Temporal variation of water-soluble species from online ACSM measurement and filter-based IC analysis during the dust influencing period (on the left). On the right, the figures show the scatter plot, and the mass concentration of each point was the mean value of the ACSM observation according to the filter sampling starting and ending times.
